# A Plea for More General Knowledge of Dental Text-Books

**Published:** 1903-04-15

**Authors:** 


### A PLEA FOR MORE GENERAL KNOWLEDGE OF DENTAL TEXT-BOOKS.

Why don't dentists read books on dentistry? Is it because to do so would be an admission that they do not know all about dentistry? We've heard that there are dentists who pretend to their patients that they know it all, and that there are others who do not. Admitting that dentists know it all (we presume that dental students have no such notions), why don't dental students read books on dentistry, or even dental journals? What is the use of demanding a matriculation that requires the student to know how to read if he will never need it after he begins the study of dentistry? We admit that it is convenient to know how to read a daily paper, but it seems wholly unnecessary for a dentist to know how to read a scientific work. Are dentists and dental students not far enough along in mental training to be able to gather information from books, or must they be shown everything before they can understand it?

Most dental students, when they go to college, buy an anatomy (usually second-hand), some buy a physiology; usually they bring with them the chemistry they used in High School. This is the sum total of the dental library some students have; and they sell it all out to the janitor when they leave college. These books (nor any other, for that matter), do not contain anything essential to a dentist in general practice. Ask a student if he has a work on dental chemistry, materia medica, orthodontia, dental pathology, metallurgy, bacteriology, or even operative dentistry, or ask him if he has read some article in a recent dental journal (which is given him free of charge), and he just smiles at you, as if to say, "Don't jolly me." If most dentists were asked if they had any of the above books of a recent edition, they would say, "I cannot fill teeth with books."

The dental book business in Canada would never make any one rich. There is only one book store in Toronto that keeps dental books, and they haven't twenty-five volumes all told. There are three or four medical book stores with thousands of volumes on their shelves. It is true there are approximately six hundred medical students to two hundred dentals, and more medical books written than dental, but the dental book is just as essential to the dental as the medical book is to the medical student, and there should not be a difference of thousands in the number kept. New books are not needed, because students sell out the few books they have when they change classes or graduate. They go out to practice, believing they know all that dental books can teach them. The one hopeful feature is that there are so many dental journals, and that they are so widely read. Medical journals are not nearly so well read by the medical profession as dental journals are by by the dental profession. Books in medicine and surgery are better written than books on dentistry, which may in a measure account for the marked difference in the number read. But if the dentists would read more books, they would create a demand, and better books would be written, and the profession would be largely the gainer.—Editorial in *Dominion Dental Journal.*

We trust the Editor has overdrawn this deplorable situation in Canada. If it is a fact then we would suggest that the Royal College of Dental Surgeons add a new admission requirement which will compel its matriculants to purchase at the outset a complete set of text-books, which he dare not dispose of until after he has graduated. The situation is not quite so desperate on this side but something might be said along the same line.—ED. REGISTER.

